# Mechanisms for redox-regulation of protein kinase C

**DOI:** 10.3389/fphar.2015.00128

**Published:** 2015-06-23

**Authors:** Susan F. Steinberg

**Affiliations:** Department of Pharmacology, College of Physicians and Surgeons, Columbia UniversityNew York, NY, USA

**Keywords:** protein kinase C, oxidative stress, tyrosine phosphorylation, Src, post-translational modifications

## Abstract

Protein kinase C (PKC) is comprised of a family of signal-regulated enzymes that play pleiotropic roles in the control of many physiological and pathological responses. PKC isoforms are traditionally viewed as allosterically activated enzymes that are recruited to membranes by growth factor receptor-generated lipid cofactors. An inherent assumption of this conventional model of PKC isoform activation is that PKCs act exclusively at membrane-delimited substrates and that PKC catalytic activity is an inherent property of each enzyme that is not altered by the activation process. This traditional model of PKC activation does not adequately explain the many well-documented actions of PKC enzymes in mitochondrial, nuclear, and cardiac sarcomeric (non-sarcolemmal) subcellular compartments. Recent studies address this dilemma by identifying stimulus-specific differences in the mechanisms for PKC isoform activation during growth factor activation versus oxidative stress. This review discusses a number of non-canonical redox-triggered mechanisms that can alter the catalytic properties and subcellular compartmentation patterns of PKC enzymes. While some redox-activated mechanisms act at structural determinants that are common to all PKCs, the redox-dependent mechanism for PKCδ activation requires Src-dependent tyrosine phosphorylation of a unique phosphorylation motif on this enzyme and is isoform specific. Since oxidative stress contributes to pathogenesis of a wide range of clinical disorders, these stimulus-specific differences in the controls and consequences of PKC activation have important implications for the design and evaluation of PKC-targeted therapeutics.

## Introduction

**FIGURE 1:**
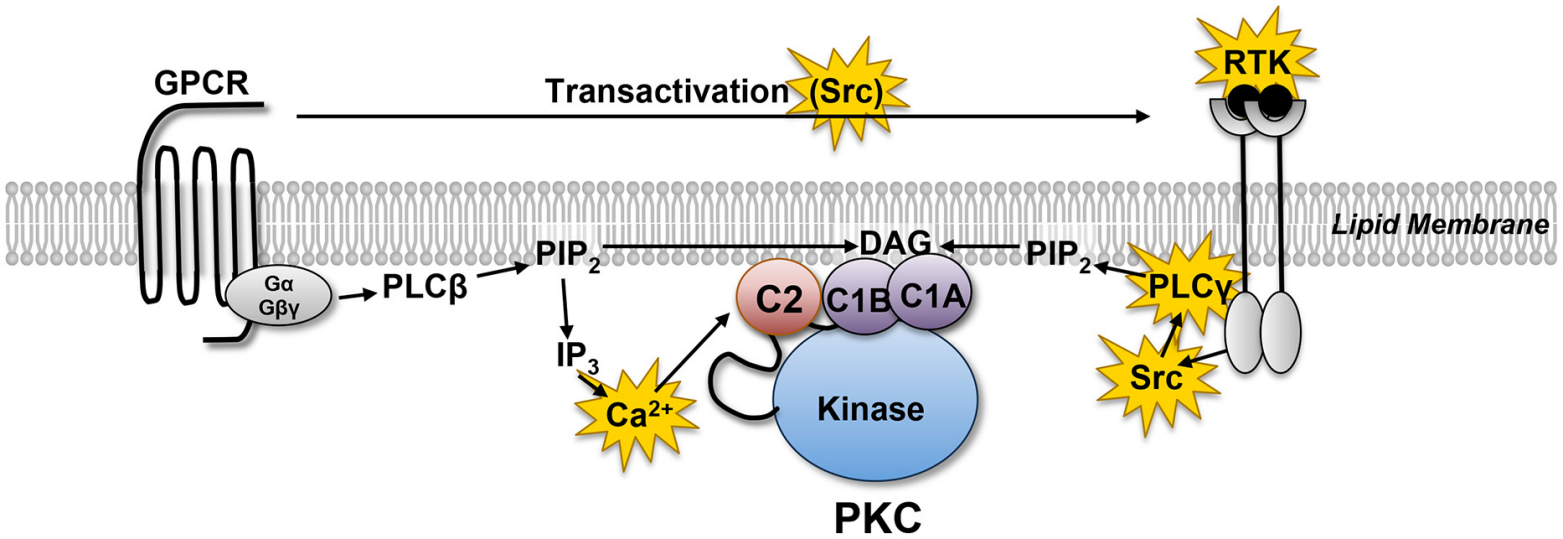
**Receptor-dependent mechanisms that allosterically activate protein kinase C (PKC).** G protein coupled receptors (GPCRs) and receptor tyrosine kinases (RTKs) activate distinct phospholipase C (PLC) family enzymes. While the regulatory controls for different PLC family enzymes differ, these enzymes act in a similar manner to hydrolyze membrane PIP_2_ leading to the generation of the calcium-mobilizing second messenger IP_3_ and DAG. The redox-sensitive elements in this pathway are highlighted in yellow (see text).

Protein kinase C (PKC) comprises a multigene family of related serine/threonine kinases that mediate a vast number of cellular signaling responses. Cardiomyocytes co-express multiple PKC isoforms that have been implicated in responses as diverse as cardiac contraction, cardiac hypertrophy, cardiac fibrosis, ischemia/reperfusion injury, and ischemic preconditioning (Steinberg, 2008, 2012). PKC isoforms were initially characterized as lipid-sensitive enzymes that are activated by growth factor receptors that stimulate phospholipase C (PLC; **Figure 1**). PLC exists as a family of enzymes with highly conserved catalytic domains that hydrolyzes phosphatidylinositol 4,5-bisphosphate (PIP_2_) to generate the calcium-mobilizing second messenger molecule inositol trisphosphate (IP_3_) and diacylglycerol (DAG), the lipid cofactor that allosterically activates PKC. PLC enzymes are subdivided into subclasses based upon the distinctive structural features in their regulatory domains and their diverse roles in cellular responses. PLCβ isoforms are activated by heterotrimeric G protein subunits (Gαq and Gβγ) and mediate G protein-coupled receptor (GPCR) responses. In contrast, PLCγ isoforms are recruited to activated receptor tyrosine kinases (RTKs) where they are activated as a result of tyrosine phosphorylation by receptor or non-receptor tyrosine kinases. Since many GPCRs transactivate RTKs, PLC-γ isoforms have been implicated in both RTK and GPCR signaling responses.

Conventional models of PKC activation focus exclusively on the second messenger-driven translocation mechanisms that deliver these enzymes in an active conformation to their membrane-delimited substrates. An inherent assumption of this model is that PKC isoforms act as generic enzymes with little-to-no substrate specificity. However, studies over the past several years have exposed additional mechanisms that alter the cofactor-requirements and/or catalytic properties of individual PKCs. Among these, the redox-dependent mechanisms that regulate PKCs are particularly interesting, since oxidative stress is a prominent feature of most clinically important cardiac disorders. While there is ample evidence that increased levels of reactive oxygen species (ROS) result in adverse cardiac remodeling and deleterious changes in contractile performance through direct redox-dependent modifications of calcium regulatory proteins and/or structural proteins in the sarcomere, this review focuses on redox-activated mechanisms that directly or indirectly influence signaling by individual PKCs. These constitute alternative mechanisms to influence pump function and/or the pathogenesis of heart failure that could provide strategies for the development of novel therapeutics.

## The Structural Features that Regulate Individual Isoforms of PKC

**FIGURE 2:**
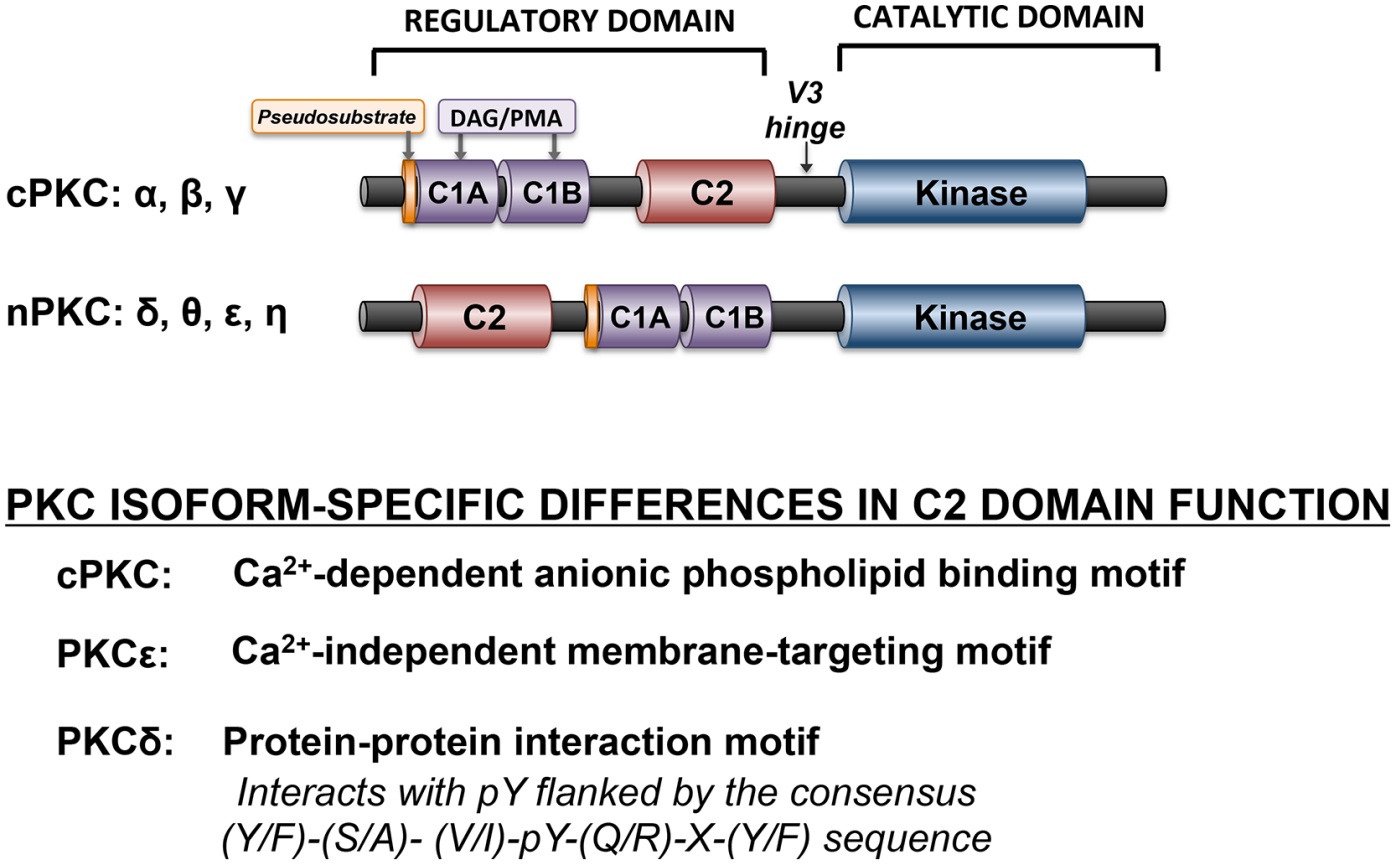
**Domain structure of cPKC and nPKC family enzymes.** The conserved pseudosubstrate motif (shown in orange) N-terminal to the C1 domain (shown in lavender), the C2 domain (red), and the kinase domain (blue) and the more variable regions (shown in black) are depicted.

Protein kinase C isoforms are structurally defined by a highly conserved C-terminal catalytic domain (consisting of motifs required for ATP/substrate-binding and catalysis) that is separated by a flexible hinge region from the N-terminal regulatory domain (**Figure 2**). Structural features in the regulatory domain provide the basis to subdivide PKCs into distinct subfamilies. Conventional PKC isoforms (cPKCs; α, the alternatively spliced βI/βII isoforms, and γ) contain two discrete membrane-targeting modules, termed C1, and C2. The C1 domain is comprised of tandem cysteine-rich C1A and C1B sequences, with a signature HX_12_CX_2_CX_13-14_ CX_2_CX_4_HX_2_CX_7_C motif (in which C is cysteine, H is histidine, and X is any residue). The six cysteine and two histidine residues in each of the twin C1 domain structures coordinate zinc ions that stabilize the zinc finger structures. C1 domains contain the binding sites for DAG and tumor-promoting phorbol esters such as phorbol 12-myristate 13-acetate (PMA). The C1 domain lipid-binding sites are surrounded by a band of hydrophobic residues that form an extended hydrophobic surface that penetrates the lipid bilayer and provides a strong driving force to anchor the C1 domains to the DAG-containing membrane. However, membrane-docking interactions based upon C1 domain binding to DAG are not sufficient to stabilize full-length cPKCs at membranes. Rather, cPKC stably associate with membranes as a result of a second membrane-targeting interaction involving the C2 domain. cPKCs C2 domains of are ∼130 residue eight-stranded anti-parallel β-sandwich structures with three inter-strand Ca^2+^-binding loops (CBLs) responsible for calcium-dependent anionic phospholipid binding.

Novel PKCs (nPKCs, δ, θ ε, and η) also have twin C1 domains and a C2 domain, although the ordering of nPKC isoform C1 and C2 domains, along the linear sequence of the protein, is switched relative to the order in cPKC (**Figure 2**). While nPKC C1 domains function as lipid-binding membrane-targeting modules (much like their counterparts in cPKCs), nPKCs are maximally activated by agonists that promote DAG accumulation or by PMA, without a calcium requirement. This difference can be attributed to a specific sequence difference between cPKC and nPKC C2 domain. nPKC C2 domains lack the critical calcium-coordinating aspartic acid residues that are highly conserved in cPKCs. As a result, the PKCε C2 domain does not bind calcium; it is a membrane-targeting module that binds lipids in a calcium-independent manner. The PKCδ C2 domain is even more distinct from a functional standpoint, as it does not bind lipids. Rather it is a protein–protein interaction domain that binds phospho-tyrosine residues flanked by the consensus sequence (Y/F)-(S/A)-(V/I)-pY-(Q/R)-X-(Y/F) (Benes et al., 2005). Recent studies indicate that the PKCθ C2 domain (which shares a high degree of structural homology to the C2 domain in PKCδ) also functions as a high affinity phosphotyrosine-binding motif (Stahelin et al., 2012). The general consensus is that PKCθ is a key signaling intermediate in immune cells and skeletal muscle, but that it is not detected in cardiomyocytes (and it has at best only a minor role in cardiovascular responses).

Regulatory regions of both cPKC and nPKC also contain a pseudosubstrate motif just N-terminal to their respective C1 domains. The pseudosubstrate motif sequence resembles a PKC substrate with an alanine substitution at the phosphoacceptor site (or P-site). For both cPKCs and nPKCs, an intramolecular autoinhibitory interaction between the regulatory domain peudosubstrate motif and the kinase domain substrate-binding pocket maintains a low level of basal catalytic activity.

## Redox-Dependent Mechanisms that Generate Lipid Cofactors that Activate PKC

Cells generate ROS through a variety of tightly controlled metabolic and cellular processes that derive from mitochondrial respiration and/or growth factor stimulation. High levels of ROS also are produced during ischemic injury or in response to many pathologic insults. There is growing evidence that the subcellular location or the chemical nature of the reactive species produced in a particular setting determine whether ROS function as second messengers in signal transduction pathways or as agents for cellular injury. A major mechanism for ROS-induced cellular regulation involves chemical modification of reactive thiols in cysteine (or to a lesser extent methionine) residues in proteins. The reactivity of a particular cysteine residue is determined by the pK_a_ of its thiol moiety; cysteine residues adjacent to basic amino acids (Arg or Lys), aromatic amino acids, or metal centers, have relatively low pK_a_’s (<6.5) and tend to be susceptible to oxidation. The resultant cysteine oxidation can lead to disulfide bond formation and conformational changes that alter protein structure and function. Since many enzymes have reactive cysteines in their catalytic centers, oxidation of these cysteine residues leads to enzyme inactivation. One important class of enzymes with extremely low pKa active-site catalytic cysteine residues is protein tyrosine phosphatases (PTPs). Since protein tyrosine phosphorylation is controlled through the reciprocal actions protein tyrosine kinases (PTKs) and PTPs – and PTPs are extremely sensitive to redox-inactivation – this tips the balance in favor of increased protein tyrosine phosphorylation. This explains PTPs strategic position as a mediator of oxidant stress-dependent signaling responses.

Oxidative stress and growth factor receptors activate many common pathways that culminate in the activation of PKCs. Two major factors explain the similarity between growth factor and redox-activated mechanisms. First, many membrane receptors couple to signaling pathways that culminate in changes in the expression and/or activity of ROS-generating or anti-oxidant enzymes. For example, GPCRs (such as angiotensin II, α_1_-adrenergic, or endothelin-1 receptors) activate signaling pathways that lead to increased ROS generation; the receptor-dependent increase in ROS in turn activates molecular targets that play critical roles in both physiologic and pathologic growth responses (Nakamura et al., 1998; Hirotani et al., 2002; Kuster et al., 2005; Guo et al., 2009).

Conversely, oxidative stress leads to the activation of certain components of the canonical GPCR- or RTK-driven signaling machinery that culminate in the activation of PKCs (**Figure 1**). For example, H_2_O_2_ treatment increases epithelial growth factor receptor (EFGR) phosphorylation and oxidation at Cys^797^, a critical residue in the active site of the kinase domain (Paulsen et al., 2012; Truong and Carroll, 2012). Recent studies establish that the direct H_2_O_2_-dependent oxidation of EGFRs at Cys^797^ increases tyrosine kinase activity (Paulsen et al., 2012). Redox-activated EGFRs recruit PLCγ, which generates the lipid cofactor DAG that activates PKCs. Of note, two other EGFR family members (Her2 and Her4) and nine other receptor or non-receptor PTKs in the human genome have cysteine residues at corresponding positions in their kinase domains, suggesting that cysteine oxidation might constitute a more common mechanism to activate a selected subset of other tyrosine kinases.

Phospholipase Cγ also is a redox-sensitive protein. PLCγ enzymes are activated when recruited via their Src homology (SH) 2 domains to phospho-tyrosine residues on activated (tyrosine-phosphorylated) RTKs. Receptor-anchored PLCγ is then tyrosine phosphorylated at critical sites that are essential to increase PLCγ activity. PLC-β isoforms do not have SH2 domains, are not regulated through tyrosine phosphorylation, and are not redox-sensitive enzymes. A number of laboratories have demonstrated that oxidative stress leads to a functionally important increase in PLCγ tyrosine phosphorylation (Wang et al., 2001). Studies of mechanism have variably attributed this to a direct redox-dependent increase in EGFR kinase activity (Wang et al., 2000), direct redox-dependent or indirect EGFR-dependent activation of Src kinases, and/or redox-dependent inhibition of PTPs. Importantly, the redox-activated PLCγ enzyme activates a cytoprotective pathway that involves PKC (Wang et al., 2001).

Src-family non-receptor tyrosine kinases (which are critically regulated through stimulatory and inhibitory tyrosine phosphorylations) also are activated through a number of redox-driven mechanisms. Src kinases are structurally characterized by regulatory SH3 and SH2 domains and a catalytic domain, that terminates in a C-terminal regulatory sequence containing an autoinhibitory tyrosine residue; Src kinases are maintained in a closed inactive conformation with low levels of basal enzyme activity as a result of an intramolecular autoinhibitory SH2 domain-C-terminal phospho-tyrosine interaction (Xu et al., 1999). Src kinases activation requires de-phosphorylation of the auto-inhibitory phospho-tyrosine (Tyr^527^ in c-Src), which releases this autoinhibitory constraint; the enzyme then autophosphorylates at a positive regulatory site in the activation loop of the catalytic domain (Tyr^418^ in c-Src). The Src SH2 domain contains cysteine residues with p*K*_a_’s low enough to be oxidized by physiological levels of ROS. Direct oxidative modification of these SH2 domain cysteine residues leads to the formation of intra-molecular disulphide bridges that secondarily disrupt the autoinhibitory Tyr^527^-SH2 domain interaction, leading to enzyme activation (Knock and Ward, 2011; MacKay and Knock, 2014). Src also contains a cysteine residue at a strategic position in its glycine-rich ATP-positioning loop (G-loop, also known as the phosphate binding P-loop). This cysteine residue (Cys^277^ in Src) also has been implicated as a target for oxidative modifications that underlie redox-dependent changes in Src kinase activity (Zhang et al., 2015). Of note, two other Src family kinases (Yes and Fgr) and four members of the fibroblast growth factor receptor family have cysteine residues at corresponding positions in their G loops. This could suggest that oxidation of a cysteine residue strategically positioned in the enzyme catalytic center constitutes a mechanism to selectively regulate only a subset of PTKs. This is in marked contrast to PTPs, which are inactivated in a more general manner through direct oxidation of a common active site cysteine. Finally, direct oxidative modification (and inhibition) of the PTP that dephosphorylate the Src activation loop site also contribute to redox-activation of Src kinase activity (MacKay and Knock, 2014). Src kinases play an essential role in the redox-dependent mechanisms that activate PKCδ, which is discussed in the Section “Direct Redox Modifications of Cysteine Residues in PKCs.”

There is limited evidence that free radicals can directly oxidize membrane phospholipids; PLC-dependent cleavage of phospholipid hydroperoxides results in the formation of diacylglycerol hydroperoxide which acts as a very potent stimulator of PKC in inflammatory neutrophils (Kambayashi et al., 2007). The relevance of oxidized lipid second messenger molecules at sites of inflammation to ischemia-reperfusion injury in cardiovascular tissues has never been considered.

## Redox-Dependent Mechanisms Involving Calcium that Can Lead to the Activation of PKC

Finally, it would be remiss to at least not mention the fact that excessive ROS can lead to calcium overload by altering the redox state of key calcium regulatory proteins. While a detailed discussion of this topic goes beyond the scope of this review [it is reviewed in Santos et al. (2011) and Tocchetti et al. (2015)], it is worth noting that several major proteins involved in excitation-contraction coupling are targets for functionally important oxidative modifications. Most studies have focused on redox modifications that target the major calcium regulatory proteins at the sarcoplasmic reticulum (SR), namely the ryanodine receptor (RyR) Ca^2+^-release channel or SR Ca^2+^-ATPase (SERCA). RyRs are macromolecular complexes comprised of four RyR protein monomers (each with a molecular mass of ∼565 kDa) bound to channel stabilizing calstabin (or FK506 binding protein) subunits and various regulatory kinases, phosphatases, and phosphodiesterases (Zalk et al., 2007). Calstabin subunits maintain RyRs in a closed state that prevents pathologic Ca^2+^ leak. Any process that triggers RyR-calstabin dissociation enhances channel open probability and increases diastolic SR calcium leak (Umanskaya et al., 2014). Recent studies indicate that RyRs contain many cysteine residues (almost two dozen per monomer) that are highly susceptible to various redox modifications and that cysteine-directed oxidative modifications of RyRs disrupt the calstabin–RyR interaction. Calstabin-dissociating redox modifications of RyRs (due to enhanced ROS generation by calcium-overloaded dysfunctional mitochondria) have been implicated in age-related skeletal muscle dysfunction (Andersson et al., 2011), abnormalities in glucose-induced insulin secretion in pancreatic β cells (Santulli et al., 2015), and disturbances in diastolic Ca^2+^ homeostasis leading to Ca^2+^ waves and ventricular extrasystoles in cardiomyocytes (Roussel et al., 2015). SERCA2a (the major SERCA isoform in cardiomyocytes) also is a target for oxidation at Cys^674^ and nitration at Tyr^294^/Tyr^295^; these oxidative modifications decrease maximal SERCA2 activity and lead to decreased calcium reuptake during diastolic relaxation. Finally, calcium overload can result from thiol oxidization of reactive cysteine residues in the pore-forming α1C subunit of the voltage-gated L-type Ca channel. This oxidative modification has been linked to increased channel activity.

Increased intracellular calcium can act as a cofactor to activate cPKCs through the conventional canonical pathway. However, increased calcium also would lead to the activation of calpain, a calcium-dependent cysteine protease that cleaves the V3 hinge region of PKCα. This proteolytic activation mechanism liberates a C-terminal constitutively active catalytic fragment (termed PKMα) that partitions to the nucleus and acts as an unregulated/mislocalized enzyme to stimulate gene programs that promote pathological structural and functional cardiac remodeling (Kang et al., 2010; Zhang et al., 2011). Of note, while PKCα is the most abundant PKC isoform in cardiomyocytes – and hence the cardiac actions of PKMα appear to predominate under conditions of calcium overload – calpain cleaves other cPKC isoforms and PKCδ (Kishimoto et al., 1989; Yamakawa et al., 2001). Hence, a calpain-dependent proteolytic activation mechanism may be a more general mechanism to activate other PKC isoforms; it may not be specific for PKCα.

## Direct Redox Modifications of Cysteine Residues in PKCs

**FIGURE 3:**
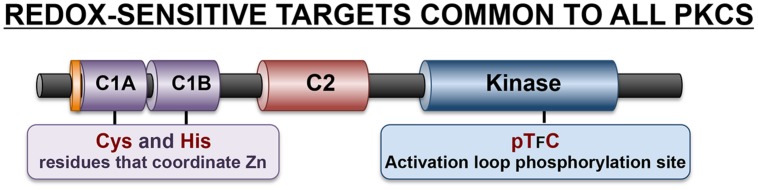
**Redox-sensitive residues in the C1 domain and kinase domain common to all PKCs**.

Both the N-terminal regulatory region and the C-terminal catalytic domains of PKCs contain cysteine residues that are susceptible to redox modifications (**Figure 3**). The cysteine residues in the regulatory C1 domain are particularly sensitive to oxidative modifications that disrupt this structure and lead to kinase activation. Studies of mechanism suggest that oxidative modification of these cysteine residues (which are precisely spaced to form a hairpin structure that coordinates zinc ions) result in the release of zinc atoms and a conformational change that disrupts the C1 domain lipid-binding pocket leading to the loss of phorbol ester binding activity (Knapp and Klann, 2000; Korichneva et al., 2002). However, the redox-activated enzyme (which does not bind phorbol esters) is nevertheless a catalytically active enzyme since the C1 domain-targeted oxidative modifications also disrupt the adjacent pseudosubstrate domain-mediated autoinhibitory intramolecular interactions that limits catalytic activity. This redox-dependent mechanism for PKC activation presumably underlies previous observations that H_2_O_2_ activates PKC without translocating the enzyme to membranes (Ohmori et al., 1998). In fact, H_2_O_2_ activates PKCs in association with a reverse redistribution of some PKC family members from the membrane to the cytosolic fraction in cardiomyocytes and certain other cell types (Goldberg et al., 1997; Rybin et al., 2004).

Oxidative modifications of cysteine residues in the C-terminal catalytic domain typically require considerably higher concentrations of H_2_O_2_ and result in enzyme inactivation. While the redox-sensitive residues in PKC kinase domains have never been localized, the redox-dependent mechanism that leads to inactivation of the catalytic subunit of protein kinase A (PKA) may be pertinent, given the high level of sequence homology between these enzymes. In the case of PKA, Cys^199^ in the activation loop is a target for *S*-glutathionylation (Humphries et al., 2002). While PKA contains a second redox sensitive cysteine at position 343 in the C-terminal variable region (that is not conserved in PKCs), there is evidence that oxidative modifications at Cys^199^ alone can be inactivating (Humphries et al., 2002, 2005). Two mechanisms have been identified. First, while Cys^199^ is not part of the catalytic mechanism, it is strategically located in in the *P* + 1 loop that interacts with protein substrates. There is some evidence that oxidative modifications at this site reduce substrate-binding affinity. Second, Cys^199^ oxidation may inhibit enzyme activity through an indirect mechanism, by facilitating dephosphorylation of the neighboring Thr^197^ residue (Humphries et al., 2005). The PKA activation loop is phosphorylated at Thr^197^ during enzyme maturation. Thr^197^ phosphorylation is a stable ‘priming’ post-translational modification that is required to structure the catalytic pocket in an optimal conformation for catalysis. PKA-Thr^197^ phosphorylation is highly resistant to phosphatases under standard experimental conditions. However, Cys^199^ oxidation renders the adjacent activation loop Thr^197^ phosphorylation site susceptible to phosphatases; this underlies redox-dependent PKA inactivation. Of note, an activation loop sequence characterized by cysteine residue strategically located at the +2 position relative to the ‘priming’ threonine phosphorylation site that is required for optimal catalytic activity also is a characteristic feature of all PKC isoforms and many other AGC family kinases (**Figure 3**). Studies of PKCα suggest that this highly conserved chemical environment is functionally important, since the oxidative state promotes PKCα activation loop site dephosphorylation (Humphries et al., 2005). These results suggest that oxidative modifications within the activation loop may play a more general role to promote the dephosphorylation and inactivation of various PKCs.

## Tyrosine Phosphorylation of PKCδ

While translocation to membranes traditionally has been considered the hallmark of PKC activation (and traditionally has been used as a surrogate marker of enzyme activation in a cellular context), this allosteric model for PKC enzyme activation by lipid cofactors has two major limitations. First, a model that focuses exclusively on lipid cofactor-mediated translocation events that deliver the enzyme to membranes assumes that the cellular actions of PKCs are exclusively membrane-delimited. In fact, PKC isoforms are recovered in mitochondria and soluble fraction of cardiomyocytes subjected to various forms of oxidative stress (Goldberg et al., 1997; Konishi et al., 2001; Juhaszova et al., 2004); PKCs in this later fraction could underlie the well-recognized actions of PKCs to regulate cardiac contractility by phosphorylating sarcomeric proteins in the contractile apparatus (at a site physically distant from the lipid bilayer). While there is some evidence that a subset of regulatory proteins associated with the contractile apparatus bind certain phospholipids (i.e., the actin capping protein CapZ interacts with PIP_2_) studies to date indicate that these lipid interactions play a structural role to regulate actin filament assemble (Russell et al., 2010). There is no evidence that sarcomeres are the site of phospholipid hydrolysis or the generation of DAG. Second, the canonical model for PKC activation views PKC’s catalytic activity as an inherent property of the enzyme that is not altered by the activation process. This model does not adequately explain the diverse (and in some cases opposing) actions of certain PKC. For example, PKCδ has been implicated in both ischemia/reperfusion injury and cardioprotection (Mayr et al., 2004; Churchill et al., 2008).

Early studies showing that PKCδ is activated in a stimulus-specific manner in various cell types provided the rationale to consider a role for oxidative stress as an alternate mechanism to activate PKCδ. Specifically, studies in cardiomyocytes established that GPCR agonists drive PKCδ to lipid-membranes membranes where it phosphorylates membrane-delimited substrates. The GPCR-dependent pathway does not lead to a detectable increase in PKCδ tyrosine phosphorylation. In contrast, oxidative stress releases PKCδ from membranes, activates Src, promotes PKCδ-Src complex formation, and Src-dependent PKCδ tyrosine phosphorylation which increases PKCδ’s catalytic activity (Rybin et al., 2004). Several other Src family kinases (including Fyn, Lyn) that are expressed by cardiomyocytes can substitute for Src in the redox-dependet pathway that activates PKCδ (Rybin et al., 2004). Importantly, the redox-dependent increases in PKCδ tyrosine phosphorylation and PKCδ activity are detected in both the soluble and particulate subcellular compartments, suggesting that tyrosine phosphorylation confers lipid-independent catalytic activity.

PKCδ contains sites for tyrosine phosphorylation throughout the structure, including in the highly conserved regulatory and kinase domains and the intervening more flexible variable hinge region. Most of these tyrosine residues are unique to the PKCδ sequence; they are not conserved in other PKC family enzymes. Early studies from the Nishizuka laboratory implicated tyrosine phosphorylation as a mechanism to regulate PKCδ catalytic activity, showing that the tyrosine-phosphorylated enzyme is a constitutively activate enzyme that no longer requires DAG as a cofactor to phosphorylate substrates (Konishi et al., 1997, 2001). It is worth noting that while the initial study from this group focused on phosphorylations at tyrosine residues in the regulatory domain (Tyr^52^ and Tyr^187^) and a series of tyrosine residues in the kinase domain [Tyr^451^, Tyr^469^, Tyr^512^, Tyr^523^ (Konishi et al., 1997)], the subsequent study concluded that a Src family kinase (in this case Lck) driven phosphorylation at Tyr^311^ (in the more variable flexible hinge region of the enzyme) mediates the H_2_O_2_-dependent increase in PKCδ activity (Konishi et al., 2001). Note: Tyr^311^ in rodent PKCδ corresponds to Tyr^313^ in human PKCδ; the remainder of this review uses nomenclature based upon the rodent PKCδ sequence. PKCδ-Tyr^311^ phosphorylation was subsequently implicated in several other PKCδ-dependent cellular responses (Lu et al., 2007; Nakashima et al., 2008). These results provided traction for the notion that a pathway that relies on tyrosine phosphorylation, rather than cysteine oxidation, underlies redox control of PKCδ activity.

Studies from our group combined molecular and biochemical approaches to implicate tyrosine phosphorylation as a key mechanism that mediates redox-dependent changes in PKCδ catalytic activity. In retrospect, our decision to use a rigorous *in vitro* kinase assay approach (which combined measurements of PKCδ autophosphorylation and PKCδ-dependent phosphorylation of many sites on heterologous substrates) proved to be essential to expose a novel mechanism for the control of PKCδ activity. We used troponin complex as substrate in the *in vitro* kinase assays since this myofilament protein complex represents a physiologically important PKCδ substrate in cardiomyocytes. Troponin complex is comprised of equimolar amounts of cardiac troponin I (cTnI), cardiac troponin T (cTnT), and cardiac troponin C. cTnI and cTnT (the “inhibitory” and ‘tropomyosin-binding’ subunits of the troponin complex) function to fine-tune calcium-dependent cardiac contractility. cTnI and cTnT both have several phosphorylation clusters that exert distinct effects on cardiac contractility. In an initial set of studies, we showed that PKCδ phosphorylates cTnI at Ser^23^/Ser^24^ when allosterically activated by lipid-cofactors; studies in skinned single cardiomyocytes linked this phosphorylation to an increase in the cross-bridge cycling rate and a reduction in myofilament calcium sensitivity (Sumandea et al., 2008). While this action of the allosterically activated PKCδ enzyme mimics the effects of the β-adrenergic receptor-PKA pathway, it is detected exclusively in our somewhat artificial reductionist model that is designed to expose mechanism; its physiologic relevance is doubtful, since the allosterically activated form of PKCδ does not localize the contractile machinery in cardiomyocytes. Rather, we identified a redox-induced Src-dependent change in PKCδ activity that is more physiologically relevant. We showed that PKCδ acquires activity toward an additional site on cTnI (it phosphorylates cTnI at both Ser^23^/Ser^24^ and Thr^144^) and it also phosphorylates a number of threonine phosphorylation sites on cTnT during oxidative stress when the enzyme is Tyr^311^-phosphorylated by Src. This change in PKCδ’s enzymology mediates the redox-dependent decrease in maximum tension and cross-bridge kinetics (i.e., a functionally distinct and physiologically relevant mechanism for PKCδ regulation of contractility (Sumandea et al., 2008)). The additional observation that the Src-dependent acquisition of cTnI-Thr^144^ and cTnT-Thr kinase activity is completely abrogated by a Y311F substitution implicated Tyr^311^-phosphorylation as the mechanism underlying the Src-dependent increase in PKCδ activity (Sumandea et al., 2008).

**FIGURE 4:**
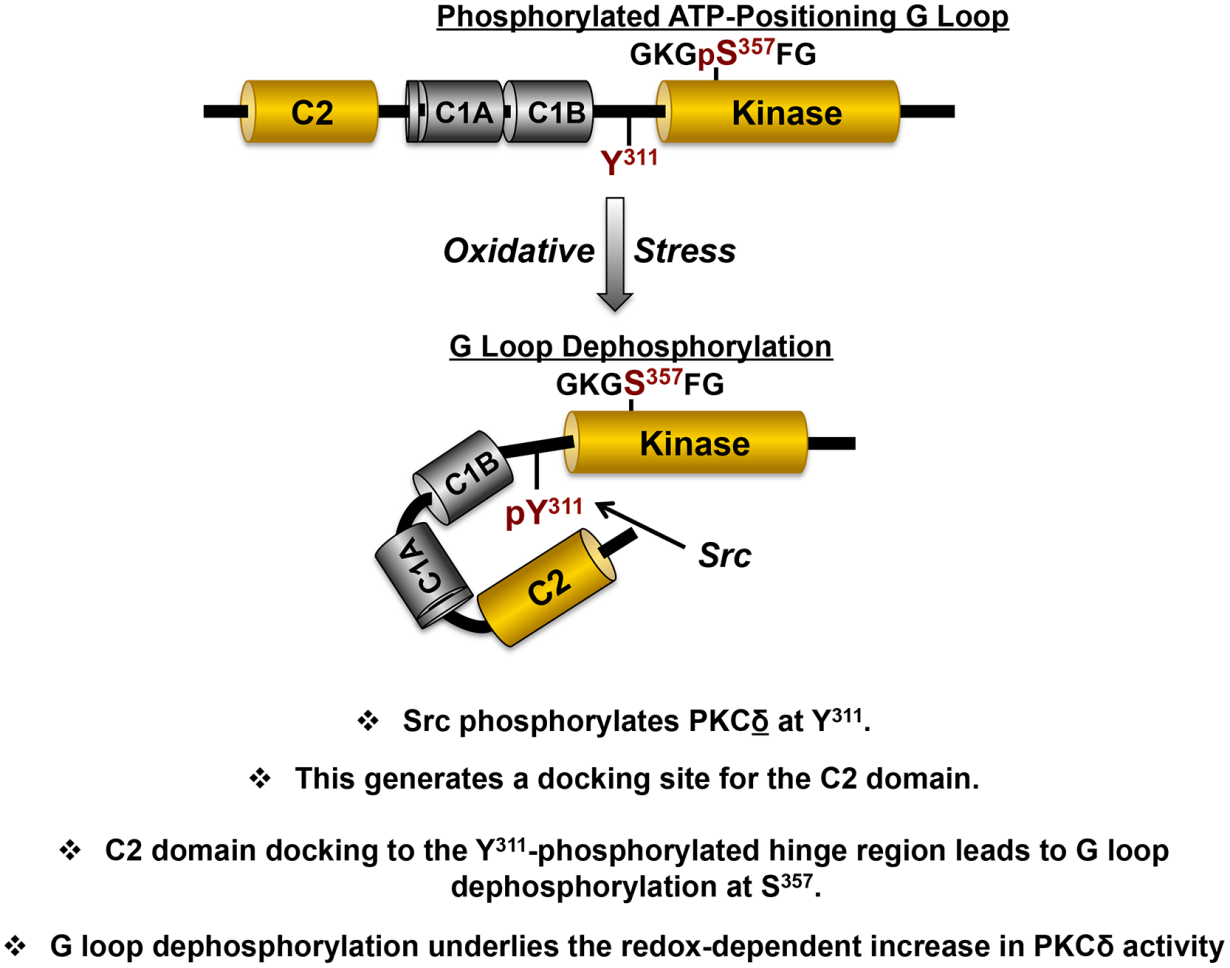
**The redox-dependent pathway involving Src that switches PKCδ from a lipid-dependent Ser kinase to a lipid independent Ser/Thr kinase.** The structural domains that mediate redox-dependent activation of PKCδ are highlighted in yellow, with the specific phosphorylation sites in the variable hinge region (Tyr^311^) and kinase domain (Ser^357^) that are key for redox-dependent activation in red (see text).

A mechanism that might explain how a phosphorylation at Tyr^311^ (in the hinge region, outside the catalytic core of the enzyme) might lead to a change in PKCδ’s enzymology is not obvious. However, we focused on the intriguing observation that PKCδ contains a phosphotyrosine binding C2 domain with a consensus binding sequence [(Y/F)-(S/A)-(V/I)-pY-(Q/R)-X-(Y/F)] that resembles the sequence flanking Tyr^311^ (Benes et al., 2005). Tyr^311^ is the only phosphorylation site in PKCδ flanked by residues that conform to a PKCδ-C2 domain consensus-binding motif (VGI-Y^311^-QGF). We used a FRET-based approach to show that PKCδ’s phosphotyrosine-binding C2 domain interacts with the Tyr^311^-phosphorylated V3 region (**Figure 4**; Gong et al., 2015). We then showed that this interaction controls PKCδ catalytic activity indirectly, by regulating PKCδ phosphorylation at a novel regulatory phosphorylation site at Ser^357^ (rodent sequence, corresponding to Ser^359^ in human PKCδ). Ser^357^ sits at the tip of the Gly-rich ATP-positioning loop (G-loop) within the small lobe of the kinase domain. The G-loop sequence is highly conserved in most protein kinases and it forms a flexible clamp that orients the γ-phosphate of ATP for transfer to substrate; localized changes in the position of this loop influence the kinetics of nucleotide binding and catalysis. We showed that PKCδ displays a high level of S^357^ phosphorylation in resting cells and that the redox-induced docking interaction between the C2 domain and the Tyr^311^-phosphorylated hinge region leads to S^357^ dephosphorylation.

Mutagenesis studies implicated G-loop dephosphorylation as a mechanism to regulate two major aspects of PKCδ’s enzymology (Gong et al., 2015). First, we showed that the PKCδ-S357A mutant is a constitutively active lipid-independent enzyme. The observation that a single non-phosphorylatable S357A substitution in the G loop confers a high level of lipid-independent catalytic activity provides a mechanism to account for the cellular actions of PKCδ in cells subjected to oxidative stress, where PKCδ phosphorylates substrates throughout the cell (not just on lipid membranes).

Second, we showed that the S357A substitution changes the enzyme’s substrate specificity (Gong et al., 2015). In way of background, most studies that define enzyme-substrate specificity focus on consensus phosphorylation motifs, residues that are either preferred or deselected at positions flanking the phosphoacceptor-site (or P-site). The literature has generally overlooked the fact that PKC isoforms (and PKA – enzymes typically characterized as Ser/Thr kinases) show a clear preference for substrates with a P-site Ser residue. In fact, our early studies showed that the allosterically activated PKCδ enzyme phosphorylates cTnI at Ser^23^/Ser^24^, but it does not phosphorylate Thr phosphorylation sites on cTnI or cTnT. The observation that the redox-activated (Tyr^311^-phosphorylated/Ser^357^ dephosphorylated) form of PKCδ acquires activity toward threonine phosphorylation sites on cTnI and cTnT provided the rationale to test whether G loop phosphorylation constitutes a mechanism to regulate PKCδ’s P-site specificity. By using a series of rigorous biochemical approaches, we were able to show that the G loop phosphorylation site – which (according to structural studies of the related PKA catalytic subunit) occupies a tight space adjacent to the P-site on substrates – regulates P-site specificity. We showed that a non-phosphorylatable S357A substitution facilitates (and a phosphomimetic S357E substitution completely abrogates) PKCδ’s Thr kinase activity. Activity toward substrates with Ser-phosphoacceptor sites is not affected. These results implicate G loop Ser^357^ phosphorylation as a novel dynamically regulated mechanism that controls PKCδ’s P-site specificity. It is worth emphasizing that a post-translational modification within the kinase domain that calibrates an enzyme’s P-site specificity in a stimulus-specific manner is unprecedented. It is tempting to speculate that the stimulus-specific differences in PKCδ phosphorylation profiles (that impact on substrate specificity) underlie PKCδ’s diverse roles in both the pathogenesis of ischemia-reperfusion injury and as a mediator of ischemic preconditioning (Steinberg, 2012). As a technical note, it also is worth noting that our studies expose an important and underappreciated caveat related to previous studies that have characterized PKCδ enzyme activity. Our studies emphasize that measurements of stimulus-dependent changes in PKCδ kinase activity are likely to differ, depending on the readout used in the assay – and particularly, the nature of the substrate (whether the substrate P-site is a Ser or Thr residue). This may explain some of the confounding inconsistencies in previous published literature.

Finally, it is worth mentioning that other tyrosine residues in the regulatory region of PKCδ, that figure prominently in the transduction of genotoxic stress responses, can also become phosphorylated in the setting of oxidative stress (Adwan et al., 2011). For example, PKCδ phosphorylation at Tyr^64^ and Tyr^155^ (sites in the regulatory domain) leads to a conformational change that exposes bipartite nuclear localization signal in the catalytic domain. This results in an interaction with importin-α, which is key for the nuclear translocation of full-length PKCδ and various PKCδ-driven proapoptotic responses. Oxidative stress also undoubtedly increases PKCδ phosphorylation at other tyrosine residues throughout the enzyme, but the controls and consequences of other redox-induced tyrosine phosphorylations (either in a cardiomyocyte context or with respect to their effects on catalytic activity) have not been interrogated. This remains fertile ground for future investigations.

## Concluding Remarks

This review summarizes our current understanding of various redox-activated mechanisms that regulate signaling by PKCs. While this narrative considers individual mechanisms separately, PKCs are subject to an elaborate and complex assortment of redox-driven modifications in various pathophysiologic settings. An understanding of the ensemble effects of these redox-driven events presents both a challenge and an opportunity for the development of novel PKC-targeted therapeutics.

## Conflict of Interest Statement

The author declares that the research was conducted in the absence of any commercial or financial relationships that could be construed as a potential conflict of interest.
